#  The Banded Elm Bark Beetle, *Scolytus schevyrewi* Semenov (Coleoptera, Curculionidae, Scolytinae) in North America: a taxonomic review and modifications to the Wood (1982) key to the species of *Scolytus* Geoffroy in North and Central America

**DOI:** 10.3897/zookeys.56.527

**Published:** 2010-09-17

**Authors:** James R. LaBonte

**Affiliations:** Plant Division, Oregon Department of Agriculture, 635 Capitol Street, Salem, Oregon, 97301-2532, U.S.A.

**Keywords:** Scolytus schevyrewi, banded elm bark beetle, exotic species, Scolytinae

## Abstract

In 2003, an Asian bark beetle, Scolytus schevyrewi Semenov (Coleoptera: Curculionidae: Scolytinae), the banded elm bark beetle, was detected for the first time in North America. This paper modifies the Wood (1982) key to the species of Scolytus Geoffroy to enable identification of Scolytus schevyrewi in North and Central America. Variation of diagnostic characters in Scolytus schevyrewi is discussed.

## Introduction

A growing number of exotic wood boring or wood associated beetles have recently been found to be established in North America (e.g., Hoebeke 1994; Hoebeke 1999; Maier and Lemmon 2000; Vandenberg et al. 2000; Mudge et al. 2001; CFIA 2002; McCullough and Roberts 2002; LaBonte et al. 2005; Haack 2006; Lee et al. 2008). In response to this trend, a multiagency pilot project to detect exotic Scolytinae throughout the United States was initiated in 2001. This program was initially designated the Exotic Forest Pest Early Detection and Rapid Response Program, but is now known as the Early Detection and Rapid Response program, or EDRR (Rabaglia et al. 2008). Since 2007, the EDRR program has been implemented on a national level under the auspices of the USDA Forest Service and, as of 2009, most states in the U.S. have been particpants.

As the cooperating taxonomist for the western states participating in the 2003 EDRR program, specimens from Lindgren funnel traps used in this survey were sent to me for identification. Early samples from the Denver, Colorado, metropolitan area contained several specimens of a species of Scolytus Geoffroy unfamiliar to me and that I was unable to key to any species in Wood (1982). I consequently sent specimens to Dr. Stephen L. Wood (deceased), Dr. Donald E. Bright (emeritus, Colorado State University, Fort Collins, Colorado) and Dr. Natalia J. Vandenberg (U.S.D.A., Agricultural Research Service, Systematic Entomology Lab, Washington, DC). These taxonomic authorities determined the specimens to be Scolytus schevyrewi Semenov, an Asian species previously unknown from North America. Shortly thereafter, I found specimens of Scolytus schevyrewi in samples from Ogden, Utah.

Subsequent trapping found this species to be abundant and clearly established in Denver and Ogden. These data stimulated extensive trapping surveysthroughout Colorado and Utah, where it was found at most sites. The realization that Scolytus schevyrewi was widely distributed in Colorado and Utah prompted several neighboring states to initiate surveys as well. At the end of 2003, Scolytus schevyrewi had been detected in twelve additional states (Negron et al. 2005). By 2008, this supposedly new exotic species had been found from coast to coast in twenty-eight states (with the earliest records from 1994), as well as in southern Canada (Lee et al. 2009). As yet, there are no records of Scolytus schevyrewi from Mexico.

## Methods

Existing taxonomic treatments for North American Scolyus (Bright 1975; Wood 1982; Furniss and Johnson 2002) do not include Scolytus schevyrewi as it was unknown from this continent when those were published. Shortly after the detection of Scolytus schevyrewi in Colorado and Utah, an image-based aid to the identification of this species was placed on the Purdue University NAPIS (National Agricultural Pest Information Site) website (LaBonte et al. 2003). This aid is more streamlined than the following modifications to the Wood (1982) key and emphasizes the differences between Scolytus schevyrewi and Scolytus multistriatus (Marsham)because the latter is much more apt to be encountered in surveys than is Scolytus piceae (Swaine). However, this early treatment is incomplete as it does not include some diagnostic characters subsequently found nor was the range of variation of some characters recognized.

The diagnostic characters used to differentiate Scolytus schevyrewi from other species of Scolytus are mainly based on specimens acquired via the EDRR project and a variety of wood boring insect surveillance programs, most funded via the USDA Cooperative Agricultural Pest Survey (CAPS) program. I have examined over 7,600 specimens of Scolytus schevyrewi from these surveys.

### Distinguishing Scolytus schevyrewi from other species of Scolytus in North and Central America

The following modifications to Wood’s (1982) key to the species of Scolytus in North and Central America will serve to identify typical specimens of Scolytus schevyrewi. Very little of the text in his key remains in the couplets below, other than the distributions, hosts, and size ranges for species dealt with therein. Several characters used in his key to discriminate among these species are not used because they are unnecessary or lead to an unduly cumbersome key, are too variable, or cannot be accurately assessed without reliably identified reference specimens, a resource many users of this key will lack. These characters can include the relative sizes of interstrial and strial punctures, whether the elytral interstriae are impressed, and subtle differences in size among abdominal sternal punctures.

**Table:** 

8(7).	Sternum 2 with base of spine touching its anterior margin (Fig. 1); posterolateral margins of sterna 2–4 each bearing small, distinct, sharply pointed tubercles (those on sternum 4 often obscured by elytra) (Fig. 2); median posterior margin of sternum 1 convex (Fig. 3); elytra unicolorous brown (Fig. 4); British Columbia and Nova Scotia to California and Florida to California and Florida; Ulmus; 1.9–3.1 mm (dorsal habitus Fig. 4, lateral habitus Fig. 5)	Scolytus multistriatus (Marsham)
–	Sternum 2 with base of spine remote from its anterior margin (Figs 6, 7); posterolateral margins of sterna 2–4 lacking tubercles (Figs 6, 8); median posterior margin of sternum 1 convex (Fig. 9) or concavely truncate (Fig. 10); in coniferous or deciduous hosts	9
9(8).	Spine on sternum 2 with base remote from posterior margin of segment (Figs 6, 7)	9a
–	Spine on sternum 2 in contact with posterior margin of segment (Figs 11, 12)	10
9a.	Spine on sternum 2 narrowly conical and sometimes pointed at apex in lateral (Fig. 6), ventral (Fig. 9), and apical (Fig. 13) views; median posterior margin of sternum 1 convex (Fig. 9); last abdominal sternum with transverse carinae distant from apex (Fig. 13); elytra unicolorous brown (Figs 14, 15); interstriae not impressed and diameter of interstrial punctures less than those of striae (Fig. 14); pronotum dark with at most very narrow pale banding along anterior and posterior margins (Fig. 15); Alaska and Nova Scotia to California and New York; Picea and, rarely, Larix; 2.2–2.8 mm (dorsal habitus Fig. 15, lateral habitus Fig. 16)	7. Scolytus piceae (Swaine)
–	Spine on sternum 2 variable, from almost absent to strongly developed, but most often broadly conical and blunt at apex in lateral view (Figs 17–20), apex often broader than base in ventral view (Fig. 10) and flattened in apical view (Fig. 21); occasional aberrant specimens with a spine on sternum 2 and a second spine on sternum 3 (Figs 22, 23); median posterior margin of sternum 1 emarginate or truncate (Fig. 10); last abdominal sternum with transverse carina very near apex (Fig. 21); elytra almost always with dark median band, with bases and apices pale (Figs 24, 25) although this band may be indistinct (Fig. 26); interstriae often impressed and diameter of interstrial punctures subequal to those of striae (Fig. 27); pronotum generally with relatively extensive pale coloration (Figs 24–26); currently known from most of the coterminous states in the U.S.A. (except for the southeastern states) and from southern Canada; deciduous hosts (known only from Ulmus in North America); 2.7–4.3 mm (dorsal habitus Figs 24–26, lateral habitus Fig. 28) (not in Wood 1982)	Scolytus schevyrewi Semenov

**Figures 1–5.**
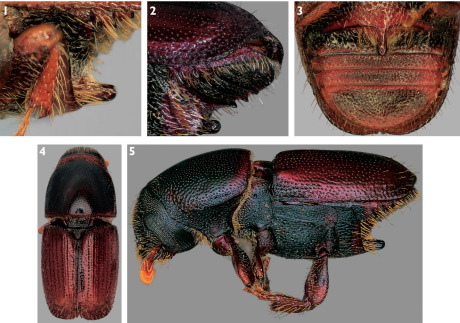
**1** Lateral view of spine on abdominal sternum 2 of Scolytus multistriatus **2** Lateral view of abdominal sterna of Scolytus multistriatus, showing lateral tubercles **3** Ventral view of abdominal sterna of Scolytus multistriatus **4** Dorsal habitus of Scolytus multistriatus **5** Lateral habitus of Scolytus multistriatus.

**Figures 6–10.**
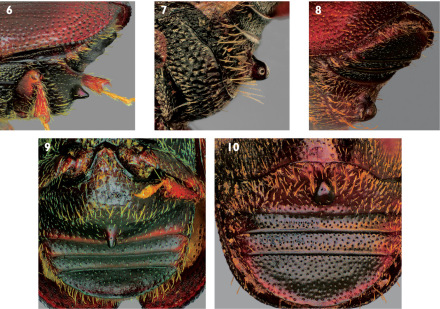
**1** Lateral view of abdominal sterna of Scolytus piceae **7** Lateral view of abdominal sterna of Scolytus schevyrewi **8** Oblique lateral view of abdominal sterna of Scolytus schevyrewi **9** Ventral view of abdominal sterna of Scolytus piceae **10** Ventral view of abdominal sterna of Scolytus schevyrewi.

**Figures 11–12.**
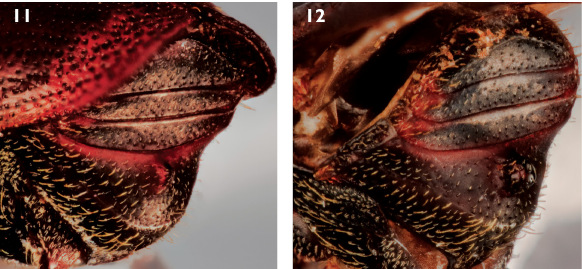
**11** Oblique lateral view of abdominal sterna of female Scolytus unispinosus LeConte **12** Oblique lateral view of abdominal sterna of male Scolytus unispinosus LeConte.

**Figures 13–16.**
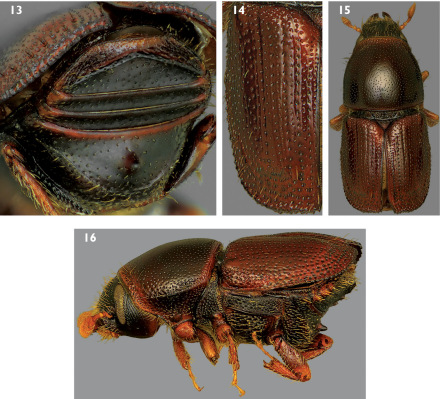
**13** Posterior view of abdominal sterna of Scolytus piceae **14** Dorsal view of left elytron of Scolytus piceae **15** Dorsal habitus of Scolytus piceae **16** Lateral habitus of Scolytus piceae.

**Figures 17–27.**
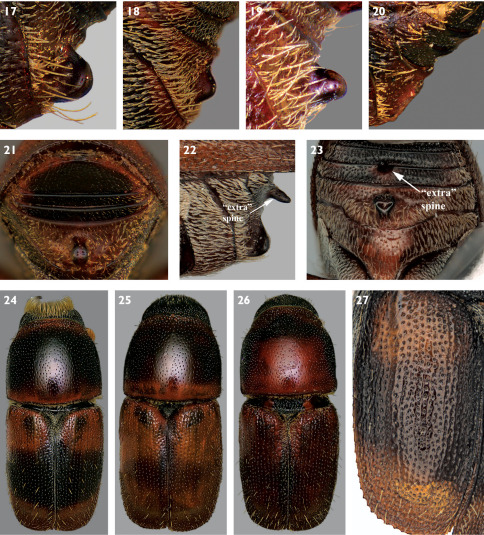
**17-20** Variation in spine on abdominal sternum 2 of Scolytus schevyrewi **21** Posterior view of abdominal sterna of Scolytus schevyrewi **22** Lateral view of abdominal sterna of Scolytus schevyrewi with two abdominal spines **23** Posterior view of abdominal sterna of Scolytus schevyrewi with two abdominal spines **24–26** Dorsal habitus and variation in elytral and pronotal coloration of Scolytus schevyrewi **27** Dorsal view of left elytron of Scolytus schevyrewi.

**Figure 28.**
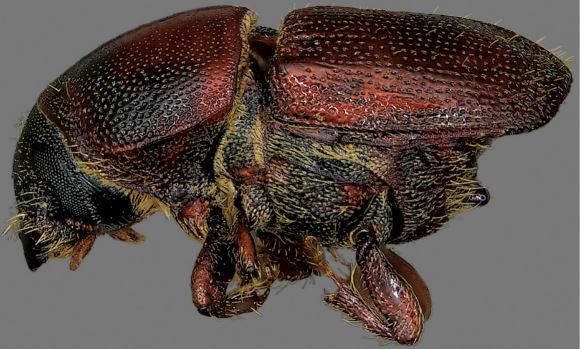
Lateral habitus of Scolytus schevyrewi.

## Diagnostic summary and character variation

Typical specimens of Scolytus schevyrewi cannot be easily confused with any other species of Scolytus known from North America. The shape and position of the spine on sternum 2 (Figs 7–8, 10, 17–21), especially in males, and typical coloration (Figs 24–26) are unique relative to all other North American species. However, this is a highly variable species and it is advisable to use a suite of characters for its identification. The following elaboration on variation in Scolytus schevyrewi is based upon the examination of thousands of specimens of that species and of Scolytus multistriatus, the species most apt to be confused with Scolytus schevyrewi.

There can be great variation in the shape and position of the spine on sternum 2. Males most often have the spine well developed, with a blunt apex that is broader than the base, appearing triangular in ventral view (Fig. 10). Especially with females, this feature can be variously reduced, becoming almost absent in the most extreme cases (Fig. 20). Reduction of the spine can lead to possible confusion with specimens of Scolytus multistriatus that have malformed or broken spines, which are not uncommon in large series thereof. However, as indicated previously, in Scolytus schervyrewi, the base of the spine on sternum 2 is remote from the anterior margin, almost at the center, whereas in Scolytus multistriatus the base of the spine is in contact with the anterior margin. Some Scolytus schevyrewi, especially those with larger spines, can have the base of the spine positioned anterior of the center of sternum 2, compounding the possibility of confusion with aberrant Scolytus multistriatus. Inadequately cleaned Scolytus multistriatus may have debris under the base of the spine, which can make its lateral appearance broader than is the case. An extreme example of abdominal spine variation in Scolytus schevyrewi is exhibited in a male from California (courtesy of R.L. Penrose, California Department of Food and Agriculture) (Figs 22, 23). In this instance, a second, accessory, sharply conical spine is present on the third abdominal sternum. This spine is asymmetrically positioned (Fig. 23), leading to my conclusion that this specimen represents a developmental aberration rather than a different species. In all other respects, this specimen appears to be a typical Scolytus schevyrewi.

The elytron of a normal Scolytus schevyrewi is distinctively colored, with a variably developed median dark band and the base and apex distinctly pale (Figs 24, 25). This character enables rapid identification of this species as no other North American species has this color pattern. The dark median band is best observed in well dried specimens; it can be obscured in specimens still damp from collecting in liquid or storage in alcohol. However, there are occasional specimens with apparently unicolorous dark or pale elytra (Fig. 26). Some samples exhibited higher proportions of these unicolorous specimens, but probably less than 10%. The existence of individuals with concolorous elytra dictates caution in complete reliance upon color for identification of Scolytus schevyrewi, as both Scolytus multistriatus and Scolytus piceae normally have concolorous elytra. Some Scolytus multistriatus also have elytra with dark apices and bases, with pale median areas.

The pronotum of a typically colored Scolytus schevyrewi is also distinctively colored, with relatively extensive areas of pale coloration (Figs 24–26). The extent of the pale areas is highly variable. The most common pattern is a fairly broad pale band along the posterior margin with a somewhat narrower pale band along the anterior margin (Figs 24, 25). Many specimens have the pale coloration extending from the posterior margin into the median area (Fig. 26). This pale coloration can sometimes cover almost the entire dorsum of the pronotum. On the other hand, in some specimens the pronotum is essentially completely dark, with only hints of anterior and posterior pale marginal banding. The extent of pale pronotal coloration appears independent of the size and extent of the median dark elytral band and the darkness of the ground color of the elytra. Several of the specimens of Scolytus multistriatus I’ve examined have large, nebulously paler areas in the median area of the pronotum. As with elytral coloration, pronotal coloration should be used with some caution to distinguish Scolytus schevyrewi from Scolytus multistriatus and Scolytus piceae.

As indicated in the key, specimens of Scolytus schevyrewi average larger than either Scolytus multistriatus or Scolytus piceae. However, small Scolytus schevyrewi fall within the size range of both of the other species. Furthermore, large Scolytus multistriatus approach the size of average or even large Scolytus schevyrewi.

Specimens of Scolytus multistriatus have distinct, pointed tubercles or “teeth” on the posterior lateral margins of sterna 2–4 (Fig. 2). Specimens of Scolytus piceae and Scolytus schevyrewi lack this feature (Figs 6, 8). This character is occasionally obscured in specimens of Scolytus multistriatus swollen with liquid preservatives, but it can normally be observed by examining the lateral margins of the sterna from an oblique perspective.

The posterior margin of sternum 2 is distinctly truncate or slightly emarginate in almost all Scolytus schevyrewi examined (Fig. 10). On the other hand, in Scolytus multistriatus (Fig. 3) and Scolytus piceae (Fig. 9) the posterior margin of sternum 2 is normally slightly to pronouncedly convex and in Scolytus multistriatus is often slightly produced at the base of the spine (Fig. 3).

The elytron of a Scolytus schevyrewi specimen in good condition typically displays three rows of relatively stout, long, discal setae that are about twice as long as the width of the elytral intervals (Figs 24–26). The elytra of most Scolytus multistriatus lack distinct rows of discal setae, although there are generally setae at the elytral apices and there may be discal setae (Fig. 4). If discal setae are present, they are generally scattered, are less stout than those of Scolytus schevyrewi and are shorter, about as long as the width of the elytral intervals. Of the small series of Scolytus piceae examined, most lacked discal setae (Fig. 14). A few specimens had a row of 3 or 4 discal setae on interval 7, but these setae were short and fine, similar to those of Scolytus multistriatus.

On visble abdominal sterna 3–5, the setae of Scolytus schevyrewi are short and recumbant (Figs 8, 10). Those of Scolytus piceae are even shorter, often difficult to discern, and are also recumbant (Figs 6, 9). In contrast, specimens of Scolytus multistriatus often have long, semi-erect setae on these sterna, especially on sternum 5 (Figs 2, 3).

In summary, a suite of characters is best used to reliably identify Scolytus schevyrewi. Especiallly with regard to Scolytus multistriatus, these include (more or less in order of reliability and ease of assessment) the shape and position of the spine on sternum 2, normal elytral and pronotal coloration, the absence of lateral teeth on sterna 2–4, average size, the truncate or slightly emarginate posterior margin of sternum 2, relatively abundant and large discal elytral setae, and short, recumbant setae on sterna 3–5.

## Discussion

The U.S. specimens collected prior to 2003, along with the extensive U.S. distribution of Scolytus schevyrewi and its great abundance in many areas, provide ample evidence that this exotic species is not new to the U.S. but is instead a legacy species that has been present for decades. Such legacy species, e.g., Xyleborinus alni (Niisima) (Mudge et al. 2001; LaBonte et al. 2005; Hoebeke and Rabaglia 2007), are probably more often the norm for newly detected exotic wood boring insects than otherwise. This is a consequence of the current surveillance technology, the limited survey efforts of the past, and the limited taxonomic expertise available to deal with the many thousands of specimens generated by current surveys.

There are profound taxonomic challenges presented by the remaining pool of undetected legacy species, truly newly introduced exotics, and the onslaught of continued new introductions as a consequence of global trade. Almost all existing taxonomic works for scolytines in North America, let alone other taxa of wood boring or wood associated insects, quite reasonably treat only those species previously known from this continent. The taxonomic infrastructure available to support surveillance for a wide spectrum of exotic wood borers has been eroding for decades and may have declined below critical and self sustaining levels. New technologies, such as extended depth of field macroscopy and LUCID™ go far to bridge this taxonomic impediment, e.g., a recent guide to the North American Siricidae (Schiff et al. 2006). Nonetheless, exotic wood boring insects, such as Scolytus schevyrewi schevyrewi, will continue to evade recognition and detection unless substantial funds and resources are devoted to expanding our taxonomic base.
